# Role of Salivary Biomarkers IL-1β and MMP-8 in Early Detection and Staging of Periodontal Disease

**DOI:** 10.3390/medicina61040760

**Published:** 2025-04-20

**Authors:** Liliana Sachelarie, Corina Laura Stefanescu, Rodica Maria Murineanu, Mircea Grigorian, Agripina Zaharia, Ioana Scrobota, Loredana Liliana Hurjui

**Affiliations:** 1 Department of Preclinical Discipline, Faculty of Dental Medicine, Apollonia University, 700511 Iasi, Romania; 2 Faculty of Medicine and Pharmacy, University Ovidius, 900527 Constanta, Romania; rodicamurineanu@gmail.com (R.M.M.); mirceagrigorian@yahoo.com (M.G.); agrizaharia@yahoo.com (A.Z.); 3 Department of Dental Medicine, Faculty of Medicine and Pharmacy, University of Oradea, 10 1st December Street, 410073 Oradea, Romania; ioana_scrobota@uoradea.ro; 4 Department of Morpho-Functional Sciences II, Discipline of Physiology, “Grigore T. Popa” University of Medicine and Pharmacy, University Street 16, 700115 Iasi, Romania; loredana.hurjui@umfiasi.ro

**Keywords:** salivary biomarkers, gingival inflammation, periodontal disease

## Abstract

*Background*: Periodontal disease is a chronic inflammatory condition affecting the supporting tissues of the teeth. Early diagnosis is crucial for preventing severe complications. This study investigates and compares the utility of two salivary biomarkers, interleukin-1 beta (IL-1β) and matrix metalloproteinase-8 (MMP-8), in the early detection and staging of periodontal disease. *Methods*: This study included 189 patients, all of whom were evaluated both clinically and biologically. Each participant provided unstimulated saliva samples, which were analyzed using ELISA methods to quantify IL-1β and MMP-8 levels. Clinical periodontal data, including probing depth (3.9 ± 0.6 mm), clinical attachment loss (3.4 ± 0.6 mm), and Plaque Index (2.35 ± 0.55), were collected for all subjects. Biomarker concentrations were then correlated with these clinical parameters and with disease severity, classified according to the 2018 EFP/AAP criteria. *Results*: IL-1β levels (210 ± 95 pg/mL) were strongly associated with early gingival inflammation, while MMP-8 levels (185 ± 140 ng/mL) correlated with periodontal tissue destruction (*p* < 0.01). MMP-8 showed a higher sensitivity for diagnosing advanced stages (*p* = 0.002), whereas IL-1β was more effective in detecting early inflammatory changes (*p* = 0.01). Logistic regression identified MMP-8 as a significant predictor of advanced disease (coefficient 0.85, *p* < 0.001) and IL-1β as an indicator of early inflammation (coefficient 0.25, *p* = 0.02). *Conclusions*: Both biomarkers are valuable tools for the diagnosis and staging of periodontal disease. MMP-8 is relevant for identifying advanced cases, while IL-1β supports early detection. Their combined use may enhance diagnostic accuracy and guide personalized periodontal care.

## 1. Introduction

Periodontal disease is increasingly recognized not only as a localized oral condition but also as a systemic health challenge due to its associations with diabetes, cardiovascular disease, and other chronic conditions [1]. These links stem from shared inflammatory pathways, positioning periodontal inflammation as a local and systemic health concern [2,3]. The early detection of periodontal disease, therefore, carries implications beyond oral health, potentially mitigating broader systemic risks through timely intervention [2,3,4].

In this context, salivary biomarkers such as IL-1β and MMP-8 offer opportunities not only for early diagnosis but also for predicting disease progression and tailoring treatment plans to individual patient profiles. Their incorporation aligns with the broader shift toward precision medicine, where interventions are guided by specific biological signatures rather than generalized protocols [5,6,7].

Gingiva, the periodontal ligament, and alveolar bone are among the supporting tissues of teeth that are impacted by periodontal disease, a chronic inflammatory condition initiated by microbial plaque buildup. This triggers an immune response that destroys periodontal tissues. If left untreated, periodontal disease can result in tooth loss and is associated with systemic conditions such as diabetes and cardiovascular disease [1,2]. Given the significant impact of periodontal health on oral and overall well-being, early diagnosis is crucial to ensure effective management and prevent complications [3].

Current diagnostic methods, such as probing depth measurements, bleeding on probing, and radiographic indicators of bone loss, are widely used in clinical practice; however, they typically detect periodontal disease only at advanced stages, when significant tissue damage has already occurred, including deep periodontal pockets, severe attachment loss, and bone resorption. These methods, although valuable for assessing disease severity, are less effective in identifying subclinical or early stages of inflammation, where intervention could prevent progression to more severe conditions. These limitations have underscored the critical need for more sensitive, precise, and non-invasive diagnostic tools that can detect the disease at its earliest stage, enabling timely intervention and a better preservation of both periodontal and systemic health [4].

Although periodontal care has advanced in recent years, traditional diagnostic methods—such as measuring pocket depth, checking for bleeding on probing, assessing clinical attachment loss, and analyzing radiographs—still have significant limitations. These methods usually detect the disease only after significant tissue damage has already occurred. They also depend heavily on the examiner’s skill, which can affect accuracy and consistency. Additionally, conventional tools do not indicate the level of inflammation or the likelihood of disease progression over time [3,4].

This is especially important because periodontal disease is not just a local problem—it has been linked to severe systemic conditions like heart disease, diabetes, complications in pregnancy, and rheumatoid arthritis. For these reasons, there is a strong need for new, non-invasive tests that can detect the disease earlier and more precisely. Salivary biomarkers offer a promising alternative. They can reflect real-time inflammation and tissue damage and may help improve both oral and overall health outcomes by enabling an earlier diagnosis and personalized treatment [4,5].

Saliva, a readily accessible biofluid, is a valuable medium for this purpose, as it contains a wide range of biomolecules, including inflammatory mediators, enzymes, and other compounds, which not only provide insights into local oral health but also reflect systemic health conditions [5].

Interleukin-1 beta (IL-1β) and matrix metalloproteinase-8 (MMP-8) are prominent salivary biomarkers that provide valuable insights into the detection and progression of periodontal disease. IL-1β, a pro-inflammatory cytokine produced by macrophages, monocytes, and neutrophils, is crucial in initiating and amplifying the inflammatory response by stimulating the production of prostaglandins and growth factors [6] levated IL-1β levels are predominantly observed in the early stages of periodontal disease and are closely associated with gingival inflammation [7,8]. In contrast, MMP-8, an enzyme from the matrix metalloproteinase family, plays a central role in collagen degradation within the extracellular matrix, directly indicating tissue destruction during advanced stages of the disease [9,10]. High salivary MMP-8 levels correlate with the severity of clinical attachment loss and the formation of periodontal pockets [11,12]. Thus, IL-1β and MMP-8 reflect distinct but complementary processes—early inflammation and structural destruction—highlighting their dual relevance for diagnosing and monitoring periodontal disease.

IL-1β is primarily produced by immune cells, such as macrophages, monocytes, and neutrophils, in response to pathogenic bacteria like *Porphyromonas gingivalis* and *Treponema denticola* [6,13]. It initiates and amplifies inflammatory responses by inducing the production of other mediators, including prostaglandins and growth factors, which contribute to connective tissue breakdown and bone resorption [13]. On the other hand, MMP-8 specifically targets collagen in the extracellular matrix, making it a direct indicator of active tissue destruction. Its elevated levels in saliva have been linked to the progression and severity of periodontitis, as well as the risk of accelerated periodontal tissue loss [11,12,14].

Combining IL-1β and MMP-8 assays has been proposed as a dual strategy for improving diagnostic accuracy. IL-1β detects early inflammatory changes, while MMP-8 evaluates tissue destruction, offering a comprehensive understanding of disease progression [15,16,17]. The early detection of elevated IL-1β levels allows for less invasive interventions, such as scaling, root planing, or targeted anti-inflammatory therapies. Elevated MMP-8 levels signal active tissue degradation, guiding more aggressive treatments like local antibiotic therapy or MMP inhibitors [11,12]. Both biomarkers have also been associated with systemic conditions like diabetes and cardiovascular diseases, highlighting the broader health implications of their modulation through periodontal treatments [11,12,13,15,16,17]. By focusing on these biomarkers, this study contributes to the growing body of research that seeks to redefine periodontal diagnostics, moving from reactive to preventive care. This approach aims to bridge the gap between clinical periodontal practice and the need for integrative, systemic health management.

This study aims to comprehensively evaluate and compare the diagnostic performance of salivary biomarkers interleukin-1 beta (IL-1β) and matrix metalloproteinase-8 (MMP-8) in identifying periodontal disease across its various stages, including health, gingivitis, and periodontitis. By analyzing the concentration levels of these biomarkers in saliva samples, we seek to determine which marker, or combination thereof, demonstrates greater sensitivity and specificity for early diagnosis.

For instance, previous research has shown that IL-1β alone exhibits an area under the curve (AUC) value of 0.88, with a 90% sensitivity and 76% specificity in distinguishing periodontitis from healthy subjects [14]. Similarly, the combination of IL-1β and MMP-8 showed an AUC of 0.84 in differentiating gingivitis from healthy individuals.

Building upon these findings, our study aims to elucidate the further individual and combined diagnostic capabilities of IL-1β and MMP-8, ultimately contributing to more effective clinical decision-making and improved patient outcomes through the development of non-invasive, saliva-based diagnostic tools.

## 2. Materials and Methods

### 2.1. Study Design

This observational, cross-sectional study included 189 patients selected from Apollonia University Dental Specialty Clinic. The study received approval from the Ethics Committee under reference number 21 on 11 November 2023. All patients were evaluated for salivary levels of both IL-1β and MMP-8 biomarkers.

Inclusion criteria were patients aged 18 to 65 years, with varying stages of periodontal disease, to assess the diagnostic capability of IL-1β and MMP-8.

Exclusion criteria: We specifically excluded individuals with known systemic conditions that could alter biomarker levels, such as inflammatory diseases, cardiovascular disorders, and diabetes. This ensured that the variations observed in biomarker levels could be attributed to periodontal disease with greater confidence, rather than to confounding systemic effects. We also excluded patients currently on anti-inflammatory or immunosuppressive medication, pregnant women, and patients with severe systemic conditions that may affect periodontal outcomes.

In addition to those with known systemic conditions, individuals undergoing orthodontic treatment during saliva collection or within the previous six months were excluded, to eliminate any potential confounding effects on biomarker levels.

This study did not account for participants’ bruxism, which may confound the association between salivary biomarkers and periodontal disease due to its potential impact on oral health.

### 2.2. Saliva Sample Collection

Unstimulated saliva samples were collected from all participants between 8:00 and 10:00 a.m. to ensure consistency and minimize the influence of circadian rhythms on salivary biomarkers. To further standardize the procedure, participants received specific pre-collection instructions: they were required to abstain from consuming any food or beverages (including water), smoking, chewing gum, or performing oral hygiene activities (e.g., brushing their teeth) for at least one hour prior to sample collection. These measures were essential to minimize contamination and variability in salivary composition caused by external factors.

During the collection process, participants were seated comfortably in a quiet environment to minimize stress, which could impact salivary flow and composition. Saliva accumulated naturally in the mouth without stimulation, ensuring samples reflected baseline conditions. Participants spat directly into sterile polypropylene containers (Nunc CryoTube Vials, Thermo Fisher Scientific, Roskilde, Denmark) pre-cooled on ice. These containers were selected for their non-reactive properties, which preserve the sample’s integrity.

Immediately after collection, the saliva samples were placed on ice and transported to the laboratory using a Thermo Scientific Nalgene Ice Bucket, which maintains temperatures between 0 °C and 4 °C, ensuring biomarker stability during transport. In the laboratory, samples were aliquoted into sterile, ice-chilled tubes (Nunc CryoTube Vials, Thermo Fisher Scientific, Roskilde, Denmark) under controlled conditions to prevent cross-contamination. The aliquots were then stored at −80 °C in an Eppendorf CryoCube F740 freezer, which ensures the long-term stability of sensitive biomolecules such as cytokines and enzymes. This low-temperature storage was critical to preserving the accuracy and reliability of the biomarker analyses.

The use of unstimulated saliva, coupled with rigorous handling and storage protocols, provided a reliable foundation for the precise quantification of IL-1β and MMP-8 levels in this study, enhancing the validity of the results.

All the collected saliva samples were carefully evaluated before being included in the study. A minimum volume of 1 mL of unstimulated saliva was required for analysis to ensure sufficient material for both biomarker measurements (IL-1β and MMP-8). Samples that did not meet this volume requirement were excluded, to maintain consistency and accuracy in the results.

Sterile polypropylene containers (Nunc CryoTube Vials, Thermo Fisher Scientific) were utilized for saliva collection. These containers were specifically chosen for their non-reactive properties, which help preserve the integrity of the samples and prevent potential interference with biomarker stability. Their design also minimizes the risk of bacterial growth during the collection and short-term storage process.

The tubes were pre-cooled on ice before collection to further ensure the samples’ quality.

All saliva samples were immediately placed on ice after collection and transported to the laboratory in a Thermo Scientific Nalgene Ice Bucket, which maintains temperatures between 0 °C and 4 °C. The samples were aliquoted under sterile conditions and stored at −80 °C until analysis to prevent enzymatic activity and bacterial proliferation.

Several potential confounding factors were considered in this study to ensure the reliability of the findings. Consistency in saliva collection was minimized by standardizing the collection protocol described above. However, individual differences in salivary flow rates and baseline salivary composition could still introduce variability. To address potential systemic influences, participants with known systemic conditions, such as diabetes, cardiovascular diseases, or inflammatory disorders, were excluded, as these conditions could significantly affect salivary biomarker levels and confound the interpretation of the results. By controlling these factors through strict inclusion and exclusion criteria, this study ensured that the observed variations in IL-1β and MMP-8 levels were attributable to periodontal disease rather than external or systemic influences.

### 2.3. Biomarker Analysis

The levels of IL-1β and MMP-8 in saliva were measured using enzyme-linked immunosorbent assay (ELISA) kits provided by R&D Systems, Inc. (Minneapolis, MN, USA). The specific kits used were the Human IL-1β/IL-1F2 Quantikine ELISA Kit and the Human MMP-8 Quantikine ELISA Kit.

Saliva samples were thawed at room temperature and vortexed to ensure homogeneity. Equal volumes of each sample were used for analysis. Standards and samples were added to 96-well plates pre-coated with antibodies specific to IL-1β or MMP-8. After incubation at 37 °C for unbound substances were washed away using an automated plate washer (BioTek ELx50, BioTek Instruments, Winooski, VT, USA). Enzyme-linked secondary antibodies were then applied, followed by a substrate solution to initiate a colorimetric reaction.

Optical density (OD) was measured at 450 nm using a Biochrom EZ Read 400 microplate reader (Biochrom Ltd., Cambridge, UK). Biomarker concentrations were calculated using standard curves generated from known concentrations provided by the ELISA kits.

This dual analysis allowed for a direct comparison of IL-1β and MMP-8 levels within the same patients, facilitating a robust assessment of their diagnostic performance.

Although IL-1β and MMP-8 were analyzed individually in some statistical comparisons, all participants were tested for both biomarkers, and no predefined grouping was used in the study design.

### 2.4. Clinical Periodontal Parameters

To correlate biomarker levels with periodontal health status, standard clinical periodontal parameters were evaluated: Plaque Index (PI—to assess oral hygiene); Gingival Index (GI—to evaluate inflammation); probing depth (PD—to measure the depth of periodontal pockets); and clinical attachment loss (CAL—to determine the extent of periodontal tissue destruction).

The clinical periodontal parameters assessed in this study included the Plaque Index (PI), Gingival Index (GI), probing depth (PD), and clinical attachment loss (CAL). The PI and GI were recorded using standardized indices widely accepted in periodontal research, employing 0–3 scales to evaluate plaque accumulation and gingival inflammation, respectively. These indices remain clinically relevant and are consistently validated in recent consensus reports and diagnostic guidelines for gingival diseases [18,19].

Probing depth (PD) was measured by millimeters at six sites per tooth using a standardized periodontal probe, such as a Williams probe. Moderate periodontal disease was defined as a probing depth of 4–5 mm, based on the American Academy of Periodontology (AAP) guidelines. Clinical attachment loss (CAL) was calculated as the distance in millimeters from the cementoenamel junction (CEJ) to the base of the periodontal pocket. Moderate attachment loss was classified as 3–4 mm, in line with the American Academy of Periodontology (AAP) definitions.

Two experienced dentists performed all the clinical measurements. To ensure reproducibility, a subset of 15% of the participants was re-evaluated by both examiners within a 48 h interval. The inter-examiner reliability was assessed using Cohen’s kappa coefficient, a statistical test that quantifies the level of agreement between two raters beyond what would be expected by chance. In this study, the Cohen’s kappa value was 0.87, indicating a high level of consistency between examiners in assessing periodontal parameters.

### 2.5. Statistical Analysis

The relationships between IL-1β and MMP-8 levels and clinical parameters were analyzed using appropriate statistical tests. Descriptive statistics, including mean and standard deviation, were calculated to summarize the data. Pearson’s correlation coefficient was used for normally distributed variables, while Spearman’s correlation was applied for non-normally distributed variables.

To assess the diagnostic relevance of IL-1β and MMP-8, their ability to distinguish between different stages of periodontal disease was evaluated. Both biomarkers demonstrated excellent diagnostic potential, with high sensitivity and specificity. All correlations and diagnostic assessments were statistically significant (*p* < 0.001).

## 3. Results

### 3.1. Baseline Characteristics

This study included 189 participants. All participants were evaluated for salivary levels of both IL-1β and MMP-8 biomarkers. The participants’ age ranged between 25 and 65 years, with a mean age of 33.5 ± 8.2 years. Table 1 presents the baseline characteristics of the participants, including age, gender distribution, education level, and residence.

### 3.2. Biomarker Levels

Table 2 presents the mean levels of IL-1β and MMP-8, measured across all the participants. The mean IL-1β level was 210 ± 95 pg/mL, while the mean MMP-8 level was 185 ± 140 ng/mL. These values reflect the broad range of inflammatory and tissue degradation responses observed in the study population. The concurrent evaluation of both biomarkers in all the participants provides valuable insights into their respective roles in the progression of periodontal disease.

### 3.3. Clinical Parameters

**Figure 1:**
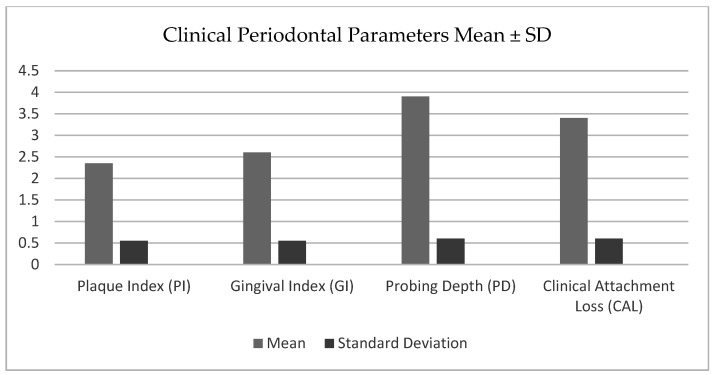
Clinical periodontal parameters.

Figure 1 provides a comprehensive overview of clinical periodontal parameters across the study groups. Each parameter offers valuable insights into different aspects of periodontal health and the underlying pathological processes.

A Plaque Index (PI) of 2.35 ± 0.55 indicates a moderate level of plaque accumulation among the participants. Plaque serves as the primary etiological factor for periodontal disease, contributing to the proliferation of pathogenic bacteria. The presence of a moderate plaque accumulation aligns with increased IL-1β levels, emphasizing its role in initiating and sustaining the inflammatory response in the gingival tissues.

A Gingival Index (GI) of 2.6 ± 0.55 reflects moderate gingival inflammation across the cohort. Elevated GI values highlight the active inflammatory processes occurring in the earlier stages of periodontal disease, driven by the host’s immune response to bacterial plaque.

A probing depth (PD) of 3.9 ± 0.6 mm suggests the presence of periodontal pockets in most participants, indicative of moderate periodontal disease. Deeper pockets are closely associated with elevated MMP-8 levels, reflecting the biomarker’s role in the degradation of the extracellular matrix. MMP-8, as a collagenase enzyme, contributes to the structural breakdown of periodontal tissues, leading to pocket formation and progression to more advanced disease stages.

A clinical attachment loss (CAL) of 3.4 ± 0.6 mm demonstrates the extent of periodontal tissue destruction in the study population. CAL is a direct measure of cumulative tissue loss, reflecting the severity of periodontal disease. Strong correlations between CAL and MMP-8 levels underscore the enzyme’s pivotal role in the degradation of connective tissue and the progression of periodontal breakdown.

By assessing IL-1β and MMP-8 levels in conjunction with clinical parameters, this study highlights the dynamic interplay between inflammatory and destructive processes in periodontal disease. IL-1β predominantly drives inflammation, as evidenced by its strong association with PI and GI, while MMP-8 plays a critical role in tissue degradation, as reflected in PD and CAL. These biomarkers collectively offer a detailed understanding of the disease’s progression, bridging the early stages of inflammation with the later phases of tissue destruction.

### 3.4. Severity of Periodontal Disease in the Study Cohort

The distribution of severity levels in the cohort is presented in Table 3.

The assessment of periodontal severity was according to the 2018 classification of stable periodontitis by the European Federation of Periodontology (EFP) and the American Academy of Periodontology (AAP). According to these classifications, periodontitis was categorized based on severity (loss of clinical attachment and radiographic bone loss) and complexity (probing depth, furcation involvement, and tooth loss). Staging was performed according to the 2018 EFP/AAP classification, where Stage I was defined by a clinical attachment loss (CAL) of 1–2 mm and probing depth (PD) ≤ 4 mm; Stage II by a CAL of 3–4 mm and PD ≤ 5 mm; and Stage III by a CAL ≥ 5 mm, a PD ≥ 6 mm, and vertical bone loss. Stage IV included all the features of Stage III, along with masticatory dysfunction, secondary occlusal trauma, or bite collapse.

The most significant proportion of patients (45.0%, 95% CI: 38.2–52.0%) fell into the moderate category, indicating a high prevalence of intermediate disease severity within the studied cohort.

### 3.5. Correlation Between Biomarker Levels and Clinical Parameters

Table 4 provides a detailed analysis of the correlations between biomarker levels (IL-1β and MMP-8) and key clinical periodontal parameters, highlighting the distinct roles of each biomarker in the progression of periodontal disease.

The correlations between salivary biomarkers and clinical parameters reveal the distinct but complementary roles of IL-1β and MMP-8 in periodontal disease progression. IL-1β levels showed a strong positive correlation with the Gingival Index (r = 0.991, *p* < 0.001), reflecting its role in driving gingival inflammation during the initial stages of periodontal disease.

Conversely, MMP-8 levels exhibited a strong positive correlation with probing depth (PD, r = 0.992, *p* < 0.001) and clinical attachment loss (CAL, r = 0.992, *p* < 0.001), emphasizing its involvement in collagen degradation and advanced tissue destruction. These findings highlight the dynamic interplay between inflammation and structural degradation: IL-1β predominantly marks early inflammatory changes, while MMP-8 indicates the severity of tissue breakdown during disease progression. The statistically significant correlations (*p* < 0.001) further underscore the potential of these biomarkers to provide a comprehensive understanding of periodontal health across different disease stages.

### 3.6. Influence of Age on Biomarker Levels

A Pearson correlation analysis evaluated the relationship between age and salivary biomarker levels. The analysis revealed a strong positive correlation between age and IL-1β levels (r = 0.986, *p* < 0.001), as well as between age and MMP-8 levels (r = 0.985, *p* < 0.001), Table 5. These results indicate that older participants exhibited significantly higher biomarker levels, suggesting an age-related increase in inflammation and tissue degradation.

### 3.7. Logistic Regression Analysis of IL-1β and MMP-8

A logistic regression analysis assessed the relationship between salivary biomarkers IL-1β and MMP-8 and the likelihood of periodontal disease, Table 6. The logistic regression analysis revealed that MMP-8 has a highly significant association (*p* < 0.01) with the likelihood of periodontal disease, particularly in advanced stages, as indicated by its higher coefficient (0.85). IL-1β, while also significant (*p* < 0.05), showed a more moderate relationship (coefficient: 0.25), supporting its role in identifying early inflammatory changes. The intercept (−1.50, *p* < 0.01) reflects a low baseline probability of disease without elevated biomarker levels.

### 3.8. Multivariate Regression Analysis of Age and Biomarkers

A multivariate regression analysis was performed to assess the influence of age on salivary IL-1β and MMP-8 levels. The results are presented in Table 7.

The results indicate that for each additional year of age, IL-1β levels increase by 4.75 pg/mL (95% CI: 4.01–5.49, *p* < 0.001), and MMP-8 levels increase by 4.33 ng/mL (95% CI: 3.58–5.08, *p* < 0.001).

A multivariate regression analysis assessed the relationship between age and salivary biomarker levels. The results indicate that for each additional year of age, IL-1β levels increase by 4.75 pg/mL (*p* < 0.001), and MMP-8 levels increase by 4.33 ng/mL (*p* < 0.001). These findings suggest that age is a significant predictor of increasing biomarker levels, further supporting the role of aging in periodontal disease progression.

## 4. Discussion

According to the findings of this study, IL-1β and MMP-8, as salivary biomarkers, may be helpful in diagnosing periodontal disease. It is worth mentioning that the great majority of patients experience periodontitis as a chronic disease characterized by tissue inflammation and destruction, which, if not managed, can result in the loss of teeth. Clinical indicators, such as probing depth or radiograph analysis, typically diagnose the disease only at its late stages, highlighting the need for non-invasive and user-friendly biomarkers [20,21].

The choice to investigate IL-1β and MMP-8 in this study was based on their complementary roles in the pathogenesis of periodontal disease. IL-1β plays a central role in initiating and amplifying the inflammatory response during the early stages of periodontal breakdown. At the same time, MMP-8 directly contributes to the degradation of collagen in periodontal tissues, indicating active tissue destruction. This dual relevance makes them highly valuable for simultaneously assessing inflammation and structural damage. Although recent research has also explored other biomarkers—such as IL-6, which has shown significant correlations with the Patient Hygiene Performance (PHP) index in crevicular fluid—we selected IL-1β and MMP-8 due to their validated use in salivary diagnostics, robust correlation with clinical indicators, and practical availability through commercial ELISA kits. Their combined analysis enhances diagnostic precision and supports early, non-invasive detection strategies in periodontal care [7,16,20].

IL-1β and MMP-8 help fill this gap by providing complementary insights into the progression of periodontal disease. The role of IL-1β in periodontal disease activity highlights the inflammatory stage, while MMP-8 represents tissue breakdown. This study demonstrated that these biomarkers correlated well with clinical criteria, including the Gingival Index (GI), probing depth (PD), and clinical attachment loss (CAL), thereby enhancing their diagnostic potential. Changes in IL-1β closely represented the initial inflammatory processes, showing a significant correlation with the average GI (r = 0.991, *p* < 0.001) [18,19]. Elevated levels of IL-1β can indicate subclinical inflammation caused by microbial plaque, which may lead to severe tissue damage if left unchecked [22].

In contrast, MMP-8, a collagen-hydrolyzing matrix metalloproteinase, is associated with more advanced disease stages characterized by excessive tissue destruction. Its levels correlated strongly with CAL and PD (r = 0.992, *p* < 0.001), confirming its role during structural destruction [22,23]. As expected, MMP-8’s enzymatic activity targets extracellular matrix components, contributing to periodontal tissue loss and pocket formation. Together, these biomarkers represent distinct yet interconnected aspects of the disease: IL-1β signals the immune system’s initial inflammatory response, while MMP-8 reflects the persistence of tissue damage and deterioration.

The comparison of these results emphasizes the varying diagnostic usefulness of IL-1β and MMP-8 across different stages of periodontal disease. Elevated IL-1β levels suggest the need for preventive measures or anti-inflammatory therapy during early stages. In contrast, increased MMP-8 levels indicate more aggressive treatment requirements, such as scaling, root planing, or adjunctive antimicrobial therapy [22,24]. Combining these biomarkers provides a more comprehensive understanding of the course of the disease, enabling clinicians to tailor diagnostic and therapeutic strategies.

The complementary roles of IL-1β and MMP-8 suggest that their combined analysis can enhance the accuracy of periodontal disease diagnostics and staging. IL-1β, being an early inflammatory marker, is particularly useful for detecting initial gingival inflammation and identifying patients at risk for disease progression. Its elevation may indicate the need for preventive interventions such as improved oral hygiene and anti-inflammatory treatments.

On the other hand, MMP-8, a key marker of tissue degradation, is more strongly associated with advanced periodontal destruction. Elevated MMP-8 levels suggest active collagen breakdown, signaling the need for more intensive periodontal therapies, such as scaling and root planing, adjunctive antimicrobial treatments, or surgical interventions in severe cases.

By integrating both biomarkers into clinical practice, IL-1β could serve as an early-warning indicator for inflammation, guiding preventive measures, while MMP-8 could help monitor disease severity and response to treatment. The results of this study demonstrate a significant association between increasing age and elevated levels of IL-1β and MMP-8. These findings are consistent with previous studies that suggest periodontal disease progression and severity tend to increase with age due to cumulative exposure to bacterial biofilms, impaired immune responses, and a reduced tissue regenerative capacity. The strong correlation observed underscores the importance of considering patient age in periodontal diagnostics and treatment planning. Future studies should explore longitudinal changes in salivary biomarkers across different age groups to further elucidate their role in disease progression [22].

Supporting this dual-biomarker approach, previous studies have highlighted the significance of IL-1β and MMP-8 in periodontal diagnostics. Kinane and Preshaw (2020) demonstrated IL-1β’s role in early inflammation and MMP-8’s role in tissue degradation, advocating for their combined use [21]. Similarly, Sorsa et al. (2021) emphasized the utility of MMP-8 in monitoring disease activity and treatment responses, particularly in cases with aggressive disease progression [11,22]. Furthermore, I-Sharqi et al. (2024) reinforced the relevance of IL-1β and MMP-8 in site-specific and whole-mouth assessments, while Cafiero et al. (2021) identified these biomarkers as highly promising for early, non-invasive screening [25,26,27,28].

Additional studies have validated the potential of salivary biomarkers in diagnosing and monitoring periodontal disease. Mohammed et al. (2024) and Blombach et al. (2023) highlighted the high sensitivity and specificity of salivary biomolecules in distinguishing disease severity and progression [29,30]. Marin et al. (2023) noted that MMP-8 could complement traditional clinical indicators for a more precise assessment of gingival and periodontal health [29,31]. Wei et al. (2023) and Liu et al. (2010) underscored the dual role of cytokines and matrix-degrading enzymes in protecting against and contributing to periodontal destruction, further supporting their integration into diagnostic workflows [32,33].

The combined application of IL-1β and MMP-8 represents a significant step forward in diagnosing and managing periodontal disease. These biomarkers enhance early identification and provide insights into disease progression and treatment outcomes. Clinicians can utilize these markers to develop stage-specific diagnostic and therapeutic plans, thereby enhancing patient care and preventing the progression to severe disease. For example, elevated IL-1β levels may indicate the need for anti-inflammatory treatments aimed at reducing early inflammatory responses, such as topical or systemic anti-inflammatory medications, improved oral hygiene practices, or adjunctive therapies, like chlorhexidine rinses [21,24]. On the other hand, a concurrent elevation in MMP-8 could signal the requirement for more aggressive interventions, including scaling and root planing, adjunctive antimicrobial treatments, or surgical therapies to address advanced tissue destruction and prevent further periodontal deterioration [22]. By utilizing these biomarkers together, healthcare providers can not only identify the stage of periodontal disease more accurately but also customize treatment strategies to the specific needs of each patient. This tailored approach has the potential to enhance therapeutic outcomes, prevent overtreatment or undertreatment, and ultimately improve long-term periodontal and systemic health outcomes. This integrative approach aligns with the principles of precision medicine, emphasizing the importance of individualized care.

Earlier studies, such as those by Sorsa et al. (2021) and Kinane and Preshaw (2020), support this strategy by demonstrating how these biomarkers capture complementary aspects of inflammation and tissue destruction [11,21,23]. Moreover, the findings of I-Sharqi et al. (2024) and Cafiero et al. (2021) underscore the potential of IL-1β and MMP-8 in predicting treatment outcomes and enhancing disease monitoring [25,26].

This study highlights the potential role of salivary biomarkers, including IL-1β and MMP-8, in assessing the severity of periodontal disease. IL-1β is more relevant for detecting early inflammatory changes, whereas MMP-8 is associated with advanced tissue destruction. Additionally, age was identified as a factor influencing biomarker levels, underscoring the need for a patient-centered approach in assessing periodontal disease.

Despite these promising findings, this study has several limitations. It was conducted at a single university dental center, which may limit the generalizability of the results. Its cross-sectional design does not allow for establishing causal relationships; longitudinal studies are needed to confirm the observed associations over time. Although patients with systemic diseases were excluded, other potential confounding factors such as smoking, socioeconomic status, oral hygiene behaviors, and genetic predisposition were not fully assessed. These aspects should be further investigated to enhance the diagnostic value and applicability of salivary biomarkers. Future research should aim to validate these findings across diverse populations and investigate the feasibility of integrating salivary biomarker analysis into routine periodontal diagnostics. While the combination of IL-1β and MMP-8 can improve early detection and personalized disease management, further studies are necessary to establish standardized diagnostic thresholds and determine their clinical applicability [19,23,24].

The findings of this study suggest that salivary IL-1β and MMP-8 could be effectively integrated into chairside diagnostic tools for the early detection and staging of periodontal disease. Their inclusion in routine screening may support timely preventive or therapeutic decisions, with IL-1β guiding early anti-inflammatory intervention, while MMP-8 indicates an active tissue breakdown and the need for more intensive periodontal treatment, thus contributing to personalized care and improved long-term outcomes.

## 5. Conclusions

IL-1β and MMP-8 serve as complementary salivary biomarkers for periodontal disease, with IL-1β associated with early inflammation and MMP-8 linked to advanced tissue destruction. The findings support the potential utility of these approaches in disease diagnostics, monitoring, and management. Further validation is required to confirm the clinical utility of these measures and to define standardized diagnostic thresholds for periodontal disease.

## Figures and Tables

**Table 1:** Baseline characteristics.

Characteristic	All Patients (*n* = 189)
Women (%)	118 (62%)
Men (%)	71 (38%)
Age Range (years)	25–65
Average Age (years)	33.5
Education (Primary/Secondary/Higher) (%)	12%/50%/38%
Residence (Urban) (%)	125 (66%)
Residence (Rural) (%)	64 (34%)

**Table 2:** Biomarker levels.

Biomarker	Mean ± SD
IL-1β (pg/mL)	210 ± 95
MMP-8 (ng/mL)	185 ± 140

**Table 3:** Severity of periodontal disease.

Severity Level	Number of Patients (n)	Percentage (%)	95% CI
Mild (Stage I)	52	27.5%	(21.2%, 34.9%)
Moderate (Stage II)	85	45.0%	(38.2%, 52.0%)
Severe (Stage III-IV)	52	27.5%	(21.2%, 34.9%)
Total	189	100%	N/A

**Table 4:** Correlation between biomarker levels and clinical parameters.

Correlation	IL-1β (r)	IL-1β (*p*-Value)	MMP-8 (r)	MMP-8 (*p*-Value)
Gingival Index (GI)	0.991	<0.001	−0.992	<0.001
Probing Depth (PD)	−0.991	<0.001	0.992	<0.001
Clinical Attachment Loss (CAL)	−0.991	<0.001	0.992	<0.001

**Table 5:** Correlation between age and biomarker levels.

Variable	Pearson Correlation (r)	*p*-Value
Age vs. IL-1β	0.986	<0.001
Age vs. MMP-8	0.985	<0.001

**Table 6:** Correlation between IL-1β and MMP-8 and the probability of periodontal disease.

Variable	Coefficient	*p*-Value	Interpretation
Intercept	−1.50	0.001	Baseline likelihood of disease without biomarkers.
IL-1β	0.25	0.020	Moderate association with early inflammation.
MMP-8	0.85	0.000	Strong association with advanced tissue damage.

**Table 7:** Regression analysis of age and biomarkers.

Variable	Coefficient	95% CI	*p*-Value	Interpretation
Intercept (IL-1β)	58.55	(50.12, 67.98)	<0.001	Baseline IL-1β level in younger participants
Age → IL-1β	4.75	(4.01, 5.49)	<0.001	Strong positive association between age and IL-1β
Intercept (MMP-8)	42.18	(35.67, 48.69)	<0.001	Baseline MMP-8 level in younger participants
Age → MMP-8	4.33	(3.58, 5.08)	<0.001	Strong positive association between age and MMP-8

## Data Availability

The original contributions presented in this study are included in the article. Further inquiries can be directed to the corresponding authors.
